# Prevalence of breastfeeding in a baby-friendly pediatric practice: an experience in Trieste, Italy

**DOI:** 10.1186/s13006-019-0239-4

**Published:** 2019-10-26

**Authors:** Mariarosa Milinco, Adriano Cattaneo, Anna Macaluso, Paola Materassi, Nicola Di Toro, Luca Ronfani

**Affiliations:** 1 Clinical Epidemiology and Public Health Research Unit, Institute for Maternal and Child Health - IRCCS “Burlo Garofolo”, Trieste, Italy; 2 Pediatric practice, Trieste, Italy

**Keywords:** Breastfeeding-friendly physician’s office, Laid-back breastfeeding, Exclusive breastfeeding prevalence

## Abstract

**Background:**

In a pediatric practice in Italy, actions were undertaken to apply the recommendations for a breastfeeding-friendly physician’s office and to promote the adoption of a semi-reclined or laid-back maternal position in breastfeeding. The aim of this study is to evaluate the effect of the actions implemented, in terms of prevalence of exclusive breastfeeding.

**Methods:**

A historical cohort study was carried out using administrative data routinely collected. All women who gave birth in 2016 and registered their newborns with the pediatric practice were included, only mothers of preterm newborns < 30 weeks gestational age were excluded. The main actions undertaken were: employment of a breastfeeding peer supporter; ensuring unlimited daily access in case of breastfeeding difficulties; provision of individual support to breastfeeding mothers in a dedicated room and advice on the laid-back position; scheduling of weekly meetings of small groups for breastfeeding support. Each infant was followed up for five months. The main study outcomes were duration of exclusive breastfeeding (only breast milk and no other liquids or solids, except for drops of syrups with nutritional supplements or medicines) and prevalence at five months.

**Results:**

A total of 265 newborn infants with a gestational age greater than 30 weeks were registered with the pediatric practice during the study period, about 18% of all infants born in Trieste in that period. Complete data were available for 252 of these (95.1%). The rate of exclusive breastfeeding at five months of age was higher than the one reported for the whole infant population of Trieste and of the Friuli Venezia Giulia Region (62.3% vs. 42.9% vs. 30.3%) in the same period.

**Conclusions:**

The implementation of breastfeeding-friendly pediatric practice and the application of laid-back breastfeeding may improve the rate and duration of exclusive breastfeeding.

## Background

Sixteen years after the accreditation of the first baby-friendly hospital in 2001, breastfeeding practices in Italy still fall short of recommendations [1]. To date (March 2018), only 26 out of more than 500 maternity hospitals are baby-friendly and only about 7% of births take place in designated maternities services [2]. The rate of initiation of breastfeeding increased from 81.1 to 85.5% between 2000 and 2013, but the mean duration was only 8.3 months and the percentage of infants less than six months of age exclusively breastfed was 42.7% (48.7% at 0–1, 43.9% at 2–3, and 38.6% at 4–5 months) [3]. These national figures conceal regional variations, with higher breastfeeding rates in the north than in the south. But even where breastfeeding rates and practices are better, the situation is far from ideal and fails to comply with the national policy recommendations of exclusive breastfeeding up to six months [1].

It is not easy to identify the causes of this gap between policy and practices. Improving hospital practices (i.e. promoting the Baby Friendly Hospital Initiative) helps increase breastfeeding rates, but not to recommended levels [4]. Even the Baby Friendly Community Initiative, introduced in Italy in 2006 [5], seems to be unable to produce the expected improvements in terms of exclusive breastfeeding rates at six months [6]. Among the many health system and social determinants of breastfeeding, research has often focused on the organization of services, on training of staff, and on support to effective latching on to the mother’s breast. Better health services and staff training, as promoted by the above-mentioned Baby Friendly initiatives (Hospital and Community), have an important but limited effect. Interventions aimed at improving positioning and latching, a core element of staff training within Baby Friendly initiatives [7], have shown to be equally ineffective [8, 9]. Some evidence is available of a possible effect on exclusive breastfeeding initiation and duration of the implementation of breastfeeding-friendly physician’s office, particularly if associated with extra care provided by a lactation consultant or by a peer counsellor [10–13].

Based on the above considerations, at the beginning of 2016 two family pediatricians (PM and AM) decided to improve the environment and the support for breastfeeding in their Trieste practice. In Trieste, a city located in the north-east Italian Region of Friuli Venezia Giulia (FVG), the Local Health Authority was accredited as Baby Friendly in 2015. Data from a cohort study carried out between 2007 and 2008 in this area showed that, at hospital discharge, 69% of infants were exclusively breastfed according to World Health Organization (WHO) definitions, and that the rate dropped to 6% by the time the infants were six months old [14]. More recently, data routinely collected by the Regional Health Authority of FVG using the same definitions [15], showed that in 2015 exclusive breastfeeding in Trieste was 43.5% at five months; factoring in also 1.7% of predominant breastfeeding, full breastfeeding amounted to 45.3%. Complementary feeding was 27.7% and formula feeding 27.0%.

To support breastfeeding in their practice, the pediatricians started by progressively applying the Academy of Breastfeeding Medicine (ABM) recommendations for a breastfeeding-friendly physician’s office [16]. They then decided to reduce the attention paid to correct positioning and latching, typical of Baby Friendly initiatives training and evaluation, and to adopt the biological nurturing method. This neurobehavioral approach to breastfeeding encourages mothers to breastfeed in a relaxed, laidback position. In this position the baby lies prone on the mother’s chest, ensuring the largest possible contact between the baby’s body and the mother’s chest and abdomen. This position opens up the mother’s body and promotes the baby’s movements through the activation of 20 primitive neonatal reflexes that stimulate breastfeeding [17–19]. The results of a small, unpublished randomized trial [20], show that, compared to standard care, the biological nurturing approach reduces the need for supplementation and the number of women who stop breastfeeding. The biological nurturing approach is simple and requires no specific position or particular procedure. To promote it, the pediatricians placed a reclining armchair in a small room and employed full-time a breastfeeding peer supporter (MM) whose role was to advise mothers on the laid-back position and to encourage them to breastfeed with no further interference, unless help was requested. This paper reports the results of that experience in terms of rate of exclusive breastfeeding and of variables associated to exclusive breastfeeding at five months.

## Methods

### Design

This historical cohort study used data routinely collected during child health visits. Data collection followed Italian regulations and laws: parents sign a standard privacy form in which they give consent to the collection and storage of child health data. The study was approved by the Regional Ethics Committee of Friuli Venezia Giulia.

### Setting

The study was carried out in a single pediatric practice located in Trieste. The practice cares for about 2000 children. Trieste is served by a third level hospital in which the mean newborn hospital stay is about three days for vaginal and five days for caesarean section deliveries. About 20 pediatric practices are present in Trieste.

### Sample

All the women who gave birth between 1 January 2016 and 31 December 2016 and registered their newborn infants with the pediatric practice were included in our analysis, only mothers of preterm newborns < 30 weeks gestational age were excluded.

According to the regulations of the Italian National Health System, new mothers choose their family pediatrician around the time their infant is born, usually in the first few days after delivery, but sometimes even before giving birth. In the absence of health problems and of breastfeeding difficulties, mothers book the first child health visit when the infant is about 30 days old, and subsequent visits are usually scheduled at three and six months. Mothers can, however, book a visit any time after registering with the pediatrician, should they have any health problem. The first child health visit normally lasts about 30 min.

In the pediatric practice where the study was performede, visits can be very frequent, even daily, in the case of breastfeeding difficulties, and can last as much as the mother wishes and needs. Mothers and infants are cared for individually in the pediatric office for clinical issues, and in the small breastfeeding room, equipped with a reclining armchair, for individual breastfeeding support provided by both the pediatricians and the peer supporter. In addition to individual care, the peer supporter meets small groups of mothers once a week in the waiting area, to discuss common concerns and share positive and negative experiences. In Italy, breastfeeding peer supporters are women with experience in breastfeeding who volunteer their time to help women with breastfeeding problems and who have attended at least a short training course, usually based on the WHO 40-h course [21]. They are not officially recognized within the national healthcare system, however, peer support for breastfeeding can be offered in hospital and outpatient settings through specific initiatives and projects. This is the case of the present study, in which the pediatric practice employed a breastfeeding peer supporter with specific training (the 20-h course on Breastfeeding Management and Promotion in a Baby Friendly Hospital, and the 40-h course on Breastfeeding Counselling, among others) and with more than 20 years of experience in supporting breastfeeding women.

Table 1 summarizes the steps recommended by the American Academy of Pediatrics for Breastfeeding Supportive Office Practices [22], in accordance with those proposed by ABM [16], and the steps implemented in the Trieste pediatric practice. In particular, all the staff of the pediatric office is trained in breastfeeding support skills with the 40-h course [21]; breastfeeding is routinely discussed with mothers at each child-health visit and women are encouraged to exclusively breastfed for six months and to continue breastfeeding as long as desired; mothers are educated on breast-milk expression before they return to work; the staff of the pediatric office collaborates with the local hospital and the community health services for matters regarding breastfeeding; the prevalence of breastfeeding is periodically monitored using routinely collected data.

**Table 1:** Summary of Breastfeeding Supportive Office Practices^a^ and steps implemented at the pediatric practice in Trieste

Steps	Steps implemented
Have a written breastfeeding-friendly office policy	No
Train staff in breastfeeding support skills	Yes
Discuss breastfeeding during prenatal visits and at each well-child visit	Yes
Encourage exclusive breastfeeding for ∼6 months	Yes
Provide appropriate anticipatory guidance that supports the continuation of breastfeeding as long as desired	Yes
Incorporate breastfeeding observation into routine care	Yes
Educate mothers on breast-milk expression and return to work	Yes
Provide noncommercial breastfeeding educational resources for parents	No
Encourage breastfeeding in the waiting room, but provide private space on request	Yes
Eliminate the distribution of free formula	Yes
Train staff to follow telephone triage protocols to address breastfeeding concerns	Yes
Collaborate with the local hospital or birthing center and obstetric community regarding breastfeeding-friendly care	Yes
Link with breastfeeding community resources	Yes
Monitor breastfeeding rates in your practice	Yes

^a^Adapted from: Meek JY, Hatcher AJ, AAP Section on Breastfeeding. The breastfeeding-friendly pediatric office practice. *Pediatrics*. 2017;139:e20170647 [22]

In the study pediatric practice, breastfeeding mothers, whether they have difficulties or not, are invited to lay back in a comfortable posture, with the baby on their chest in ventral position, after a brief explanation on the primitive maternal and neonatal reflexes that facilitate a good latch and on the positive use of gravity. Mothers are then left undisturbed in order to avoid any interference with their instinctual behaviors. In particular, both the pediatricians and the peer supporter avoid any form of teaching on how to breastfeed using a hands-off approach. When the result of this initial phase is successful, mothers are discharged with the recommendation to apply the same laid-back position at home, when they consider it helpful. In case of difficulties, mothers are given additional advice and support and are referred for a further session of laid-back breastfeeding one or two days later, and again subsequently, until a good latch is established.

### Outcomes

The main outcome of interest was the prevalence of exclusive breastfeeding at five months, defined according to the WHO as infants receiving only breast milk, from their mother or from a wet nurse, through breastfeeding or breast milk expression, and no other liquids or solids, except for drops of syrups with nutritional supplements or medicines; according to WHO, the “complementary feeding” category includes infants receiving breast milk and other food or liquid, including non-human milk and formula [23]. The 24-h recall period recommended by WHO was used [24]. Breastfeeding rates were evaluated also at discharge from the maternity ward, and at one and three months of age.

To study the association between possible explanatory factors and exclusive breastfeeding at five months, the following variables were considered: mother’s nationality (Italian vs. non-Italian), age (< 29; 30–39; ≥ 40 years), education (primary/intermediate vs. secondary/higher), occupation (employed vs. non-employed), type of delivery (vaginal vs. caesarean section), parity (primiparity vs. multiparity), gestational age at delivery (< 37 vs. ≥ 37 weeks), single pregnancy (yes vs. no), birthweight (< 2500 vs. ≥ 2500), time at first visit (≤ 30 vs. > 30 days), and breastfeeding at discharge from the maternity ward (exclusive vs. other).

Data on parents and infants were recorded during routine child health visits using the custom software and database employed by all family pediatricians in the region. Each infant was followed up to at least five months of age. Data on breastfeeding in the maternity hospitals were derived from the discharge letters. The remaining data on breastfeeding were collected during the routine child health visits at one, three and five months of age.

The prevalence of exclusive breastfeeding at five months in the pediatric practice was compared with the one reported in the same period by the FVG health information system for the whole infant population in Trieste and in FVG. In FVG data on prevalence of exclusive breastfeeding were routinely collected at birth and at five months of infant’s age (second immunization visit) using the same definitions and methods recommended by the WHO [15].

### Statistical analysis

Continuous variables are reported as median and interquartile range (IQR); categorical data as number and percentage. To compare the descriptive data and the prevalence of exclusive breastfeeding at five months between the sample of children of the pediatric practice and the general population of Trieste and of the FVG Region, the binomial probability test was used. Chi-square (or the Fisher’s exact test when appropriate) was used to test the bivariate association between possible explanatory variables and exclusive breastfeeding at five months. Variables associated with *p* <  0.05 were subsequently entered into a logistic regression model (forward stepwise). The statistical analysis was performed using IBM SPSS Statistics for Windows, Version 23.0. Armonk, NY: IBM Corp.

## Results

A total of 265 newborn infants with gestational age greater than 30 weeks were registered with the two family practice pediatricians in 2016. This amounts to about 18% of all the infants born in Trieste in that period. Table 2 shows some characteristics of the 252 (95.1%) mother and baby dyads with complete data, and the comparison with the last official data available for the general population of newborns of the FVG region (2011–2013 period) [25]. A statistically significant difference between the two population was seen for: the proportion of non-Italian mothers and fathers, the proportion of primiparity, the proportion of mothers between 20 and 29 years of age and 40 years or older, and the proportion of children with birthweight ≥4000 g. Most infants, 67.5%, were seen for the first time when they were less than one month of age. In particular, 28.2% were seen during their first two weeks of life, in most cases because of mothers reporting breastfeeding difficulties.

**Table 2:** Characteristics of the study population (*N* = 252) and comparison with the last official data available for the general population of newborns of the FVG region

Characteristic n (%)	Trieste pediatric practice (*n* = 252)	FVG Region 2011–2013 (number of births = 28,916)	*p* value*
Mother not Italian	81 (32.1)	6762 (23.4) (32 missing)	0.002
Father not Italian	72 (28.7) (1 missing)	5527 (19.4) (443 missing)	< 0.001
Maternal education			
Primary/intermediate	48 (19.0)	6496 (22.5)	0.20
Secondary/higher	204 (81.0)	22,404 (77.5) (16 missing)	
Paternal education			
Primary/intermediate	65 (25.9)	8198 (29.8)	0.17
Secondary/higher	186 (74.1) (1 missing)	19,334 (70.2) (1384 missing)	
Mother employed	168 (66.7)	19,493 (67.4) (16 missing)	0.79
Maternal age			
Less than 20 years	2 (0.8)	315 (1.1)	1.00
20–29 years	62 (24.6)	8867 (30.7)	0.04
30–39 years	145 (57.5)	17,476 (60.5)	0.33
40 years and more	43 (17.1)	2242 (7.8) (16 missing)	< 0.001
First child	181 (71.8)	15,205 (52.6)	< 0.001
Cesarean-section	54 (21.4)	6735 (23.3)	0.55
Gestational age			
< 31 weeks	–	233 (0.8)	Na
31–36 weeks	19 (7.5)	1706 (5.9)	0.28
37–42 weeks	233 (92.5)	26,974 (93.3)	0.61
> 42 weeks	0	3 (0.01)	0.1
Twin delivery	6 (2.4)	469 (1.6)	0.31
Birthweight^§^			
Less than 2500 g	23 (9.1)	1991 (6.8)	0.17
2500–3999 g	221 (87.7)	25,422 (86.5)	0.65
4000 g and more	8 (3.2)	1978 (6.7) (2 missing)	0.02
Age first visit			
15 days or less	71 (28.2)	Not available	
16–30 days	99 (39.3)		
More than 30 days	82 (32.5)		

* binomial probability test, two sided

^§^ Data for the FVG Region are referred to newborns (*n* = 29,393)

**Fig. 1:**
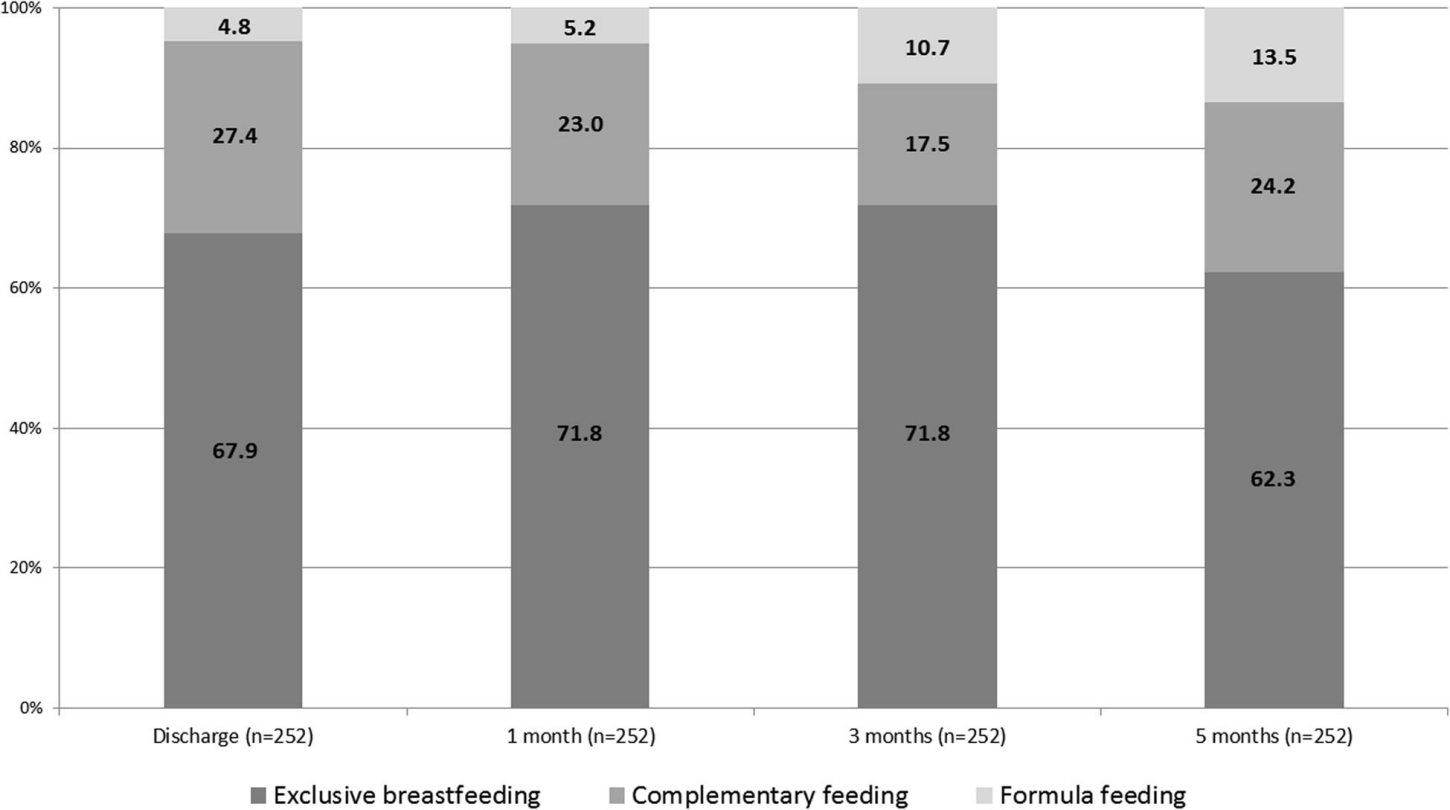
Types of feeding at different ages

Figure 1 shows the rates of exclusive breastfeeding and of other types of feeding at discharge and at one, three and five months for the 252 mother and baby dyads with complete data. Data on the age of the infants at the three collection points are shown in Table 3. Some mothers, who were discharged from the maternity hospital with complementary feeding, recovered exclusive breastfeeding at one month. Exclusive breastfeeding remained high at three months and started dropping at five. Up to the age of three months, complementary feeding is a combination of breastmilk and formula, from the age of five months it includes also complementary foods.

**Table 3:** Infants’ age in days, at routine child health visits

Child health visit	Median (interquartile range)
First month	31.0 (26.0–35.8)
Third month	92.0 (87.0–100.0)
Fifth month	159.0 (153.0–164.8)

At five months the rate of exclusive breastfeeding of children followed up in the pediatric practice was statistically significantly higher than the one reported in the same period for the whole infant population of Trieste and of FVG (62.3% vs. 42.9%, *p* <  0.0001 and vs. 30.3%, *p* <  0.0001, respectively).

At bivariate statistical analysis, a non-statistically significant higher rate of exclusive breastfeeding at five months was seen in non-Italian mothers (65.4% vs. 60.8%), in women with higher education (63.7% vs. 56.3%), in non-working women (65.5% vs. 60.7%), in mothers who had a vaginal delivery (63.6% vs. 57.4%), and in multipara (66.2% vs. 60.8%). A statistically significant associations with exclusive breastfeeding at five months was seen for mothers aged 30–39 years compared to those < 29 years and ≥ 40 years (69.0% vs. 56.3% vs. 48.8, respectively; *p* = 0.04), infants 37–42 weeks of gestational age compared to those less than 37 weeks (65.2% vs. 26.3%; *p* = 0.001), singletons compared to twins (64.4% vs. 23.1%; *p* = 0.006, Fisher’s exact test), infants with birthweight greater than 2500 g compared to those with lower birthweight (66.8% vs. 17.4%; *p* <  0.0001), infants seen for the first time after 30 days of age compared to those seen earlier (74.4% vs. 56.5%; *p* = 0.006), and exclusive breastfeeding vs. other at discharge from the maternity ward (76.6% vs 32.1%, *p* <  0.001).

The logistic regression model shows that three variables have a positive association with exclusive breastfeeding at five months: birthweight 2500 g or more (Odds Ratio [OR] 7.8; 95% Confidence Interval [CI] 2.1, 28.2), exclusive breastfeeding at discharge from the maternity hospital (OR 5.4; 95% CI 2.9, 10.0), and first visit after 30 days of age (OR 3.4; 95% CI 1.6–7.0), meaning that infants in these categories have almost 8, more than 5 and more than 3 times, respectively, higher odds of being exclusively breastfed at five months compared to the infants with birthweight less than 2500 g, non-exclusive breastfeeding at discharge from the maternity hospital, and seen for the first time before 30 days of age.

## Discussion

The data gathered during the implementation of the protocol for a baby-friendly pediatric practice and of the support of breastfeeding using the biological nurturing approach, show that the rate of exclusive breastfeeding that can be achieved at five months of age is much higher than the one reported in the same period for the whole infant population of Trieste and of FVG by the Regional Health Authority (unpublished) using the same definitions and methods recommended by WHO (62.3% vs. 42.9 and 30.3%, respectively). It is higher than the 27% (range among Local Health Authorities: 10 - 45%) recorded in 2012 in Lombardia, where the rate of exclusive breastfeeding at discharge was similar (67.3%) [26]. It is higher than the rate of full breastfeeding reported from Emilia Romagna in 2015: 33% (range among Local Health Authorities: 26 - 46%), of which only 27% was exclusive [27]. It is also higher than the rate estimated for the whole of Italy, which is probably close to the 38.6% reported at 4–5 months of age in 2013, as mentioned in the introduction. In fact, our exclusive breastfeeding rate at five months, based on WHO definitions and 24-h recall, is probably one of the highest recorded in the European Region of WHO; a compilation of national data published in 2016 reports a figure of 49.3% at six months from Slovakia, while reports from all the other member states range from 1 to 43.9% [28].

It is obviously difficult to establish a cause and effect relationship between our intervention and the observed high rate of exclusive breastfeeding, as is assessing the individual contribution of the actions implemented by the two pediatricians in their practice: promotion of the baby-friendly pediatric practice protocol, and the adoption of the biological nurturing approach, including the extra care provided by the peer supporter. Our study is purely observational and was carried out using data routinely collected by the two pediatricians. We did, however, include a non-selected population of mother-infant pairs: only preterm newborns < 30 weeks, which account for about 1% of all births in Trieste, were excluded. This exclusion does not explain the higher rates of exclusive breastfeeding found in our population. Indeed, the comparison with the last available administrative data on births in FVG (Table 2) shows that the prevalence data of low birthweight, of births 31 to 36 weeks of gestational age, and of twin deliveries, conditions usually associated with reduced rates of exclusive breastfeeding, were higher in our population [25].

There is some evidence from similar studies that a breastfeeding friendly pediatric practice may have a positive effect, regardless of the presence of a lactation consultant or of a peer supporter. In Rio de Janeiro, Brazil, the prevalence of exclusive breastfeeding among infants aged four to six months rose from 41 to 82% between 2001 and 2004, after a basic health center was accredited as breastfeeding friendly [10]. The application of the ABM protocol for a breastfeeding-friendly physician’s office in two community practices in Northern Virginia, USA, resulted in statistically significant increases in the rates of exclusive breastfeeding at two, four and six months in a before-and-after comparison [11]. Baby-friendly changes in a pediatric practice with a lactation consultant helping mothers with breastfeeding difficulties, led to an increase in non-formula feeding in Cleveland, Ohio, USA, between 2007 and 2009 [12]. It is well known that peer support effectively improves the rates of breastfeeding initiation, duration, and exclusivity [13]. The three studies cited above and the review on peer counselling lend support to the hypothesis that a breastfeeding-friendly pediatric practice may help increase breastfeeding rates, especially when extra care is provided by a lactation consultant or by a peer counsellor [10–13].

What is more difficult to support with convincing evidence is the hypothesis that the adoption of the biological nurturing approach may yield further benefits in terms of exclusive breastfeeding. Most of the available evidence derives from the observation of the physiology of breastfeeding, i.e. of the presence in all healthy mother and newborn dyads of innate reflexes aimed at initiating and establishing breastfeeding, if left undisturbed in a comfortable semi-reclined position, with the baby placed ventrally on the mother’s chest [18, 19]. There is very little literature, if any, on the effect of the biological nurturing approach on breastfeeding rates. Recently, a small, unpublished, randomized controlled trial was carried out in France for a doctorate in human lactation [20]. In this study, 32 mother and infant dyads with latch-on problems in the first two days after birth were randomized to laid-back breastfeeding or standard support. Infants in the laid-back breastfeeding group had significantly fewer formula supplements (19% vs. 26%) and none of the mothers in this group stopped breastfeeding during the first week compared with nine in the standard support group.

As expected, a birthweight of 2500 g or more and exclusive breastfeeding at discharge from the maternity hospital, were associated with a higher prevalence of exclusive breastfeeding at five months. The third variable associated with exclusive breastfeeding at five months was the time of first visit > 30 days after birth. A possible explanation for this result is that mothers who weren’t experiencing problems with breastfeeding, rarely scheduled the first child health visit before 30 days after birth.

Furthermore, we cannot exclude a selection bias due to the fact that our pediatric practice may have attracted women with an interest in breastfeeding, given the support offered by the pediatricians and the peer supporter. However, this seems unlikely for the following reasons: in Italy, families choose to enroll with a pediatric practice mostly based on the catchment area and on the number of patients already registered; the presence of a peer supporter was not advertised; the socioeconomic characteristics of the study population (maternal and paternal education and employment) were comparable with those of the general population of the FVG Region, except for a higher proportion of foreign families in our practice.

## Conclusions

Our study contributes some evidence to the already well established recommendations of the ABM for implementing breastfeeding-friendly pediatric practice [16]. More research is needed to support the hypothesis that the application of laid-back breastfeeding may contribute to increasing rate and duration of exclusive breastfeeding.

## Data Availability

The dataset used during the current study is available from the corresponding author on reasonable request.

## Abbreviations

ABM: Academy of Breastfeeding Medicine
FVG: Friuli Venezia Giulia Region
IQR: Interquartile range
OR: Odds Ratio
WHO: World Health Organization
